# A Case of Treatment With Dabigatran for Cerebral Venous Thrombosis Caused by Hereditary Protein C Deficiency

**DOI:** 10.7759/cureus.15473

**Published:** 2021-06-06

**Authors:** Taiki Fukushima, Yoshimitsu Shimomura, Satomi Nagaya, Eriko Morishita, Osamu Kawakami

**Affiliations:** 1 Neurology, Anjo Kosei Hospital, Anjo, JPN; 2 Hematology, Kobe City Medical Center General Hospital, Kobe, JPN; 3 Clinical Laboratory Sciences, Kanazawa University, Kanazawa, JPN

**Keywords:** cerebral venous thrombosis, protein c deficiency, direct oral anticoagulants, dabigatlan, magnetic resonance venography

## Abstract

A 37-year-old woman was admitted to our hospital with involuntary movements. She had no medical or family history of thromboembolism, nor was she on any medication. She showed no impaired consciousness, cranial nerve abnormalities, abnormal breathing, stiff neck or paralysis. Magnetic resonance venography exhibited poor visualization of intracranial vein. The protein C activity level reduced but the protein C antigen level was normal. Genetic analysis revealed a heterozygous mutation in exon 7 c.577-579delAAG, p.Lys193del on protein C gene. She was diagnosed with cerebral venous thrombosis and hereditary protein C deficiency type II. She received heparin in acute phase, and switched to dabigatran in chronic phase. Consequently, she had no recurrence of cerebral venous thrombosis and other complications. Dabigatran might be one of the alternative choices for patients with cerebral venous thrombosis and protein C deficiency.

## Introduction

Cerebral venous thrombosis is a rare disease that accounts for 0.5% of all strokes and is often caused by congenital thrombotic predisposition, oral contraceptives, and infectious diseases [1]. Among congenital thrombotic predispositions, hereditary protein C deficiency is a rare genetic disorder that causes various thrombosis [2]. The incidence of inherited protein C deficiency is 0.2%-0.5 % in the healthy general population [3] and 2%-5 % in individuals with venous thromboembolism [4]. Protein C deficiency can be subdivided according to whether the deficient activity is due to reduced protein levels (type I) or to reduced protein function (type II). Particularly in protein C deficiency type II, careful differential diagnosis is required because several other diseases, such as infections and liver diseases, can reduce protein C activity. Since these diseases are both rare, there have been only a few coherent reports of the cerebral venous thrombosis caused by protein C deficiency. However, the standard treatment for cerebral venous thrombosis caused by protein C deficiency is the administration of heparin in the acute phase [1]. Additionally, continuous anticoagulant use is required to prevent the development of thromboembolic events in the chronic phase [5,6]. Conventionally, warfarin is considered a standard anticoagulant but can produce some problems in general and specific problems in protein C deficiency patients, such as warfarin-induced skin necrosis [7,8]. Here, we report a rare case of cerebral venous thrombosis caused by protein C deficiency treated with dabigatran.

## Case presentation

**Figure 1:**
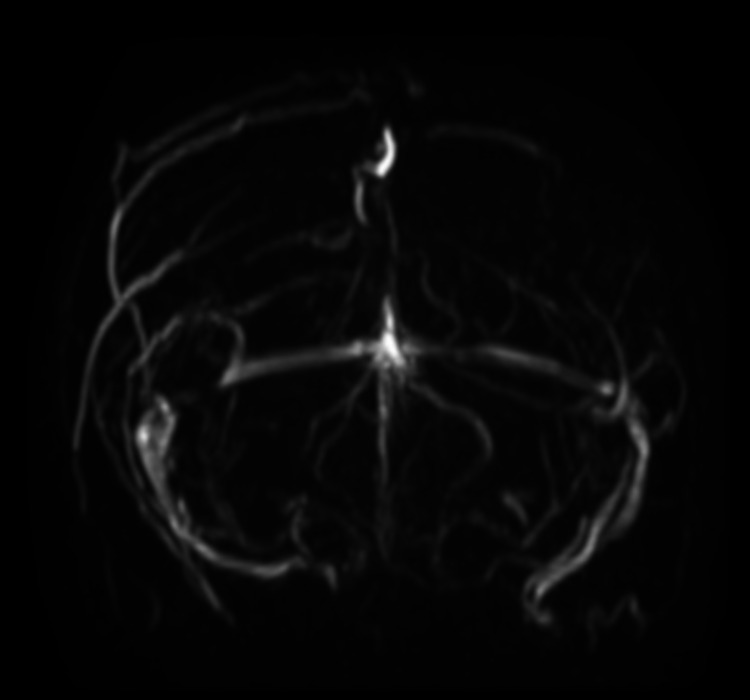
Brain magnetic resonance imaging (MRI) on admission. Magnetic resonance venography revealed poor visualization of the superior sagittal sinus, right lateral sinus, and bilateral cortical vein.

A 37-year-old Filipino woman presented to our hospital with involuntary movements of her right upper and lower limbs. She had no medical or family history of thromboembolism, nor was she on any medication. She showed no impaired consciousness, cranial nerve abnormalities, abnormal breathing, stiff neck or paralysis. Complete blood count and coagulation tests showed hemoglobin 9.1 g/dl; platelet count, 24.7 × 104/μl; white blood cell count, 10,200/μl, prothrombin time-international normalized ratio, 1.16; and activated partial thromboplastin time ratio 0.97. Magnetic resonance venography exhibited poor visualization of the superior sagittal, right lateral, and bilateral cortical veins (Figure 1).

**Figure 2:**
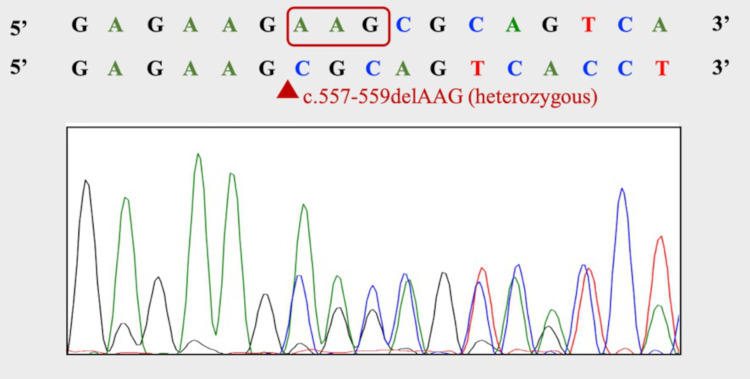
A genetic analysis. A genetic analysis revealed heterozygous mutation at exon 7c. 577-579delAAG, p.Lys193del on protein C.

Magnetic resonance imaging showed low T2 * signal in the cortical veins in both the parietal regions. Based on the imaging findings, the patient was diagnosed with cerebral venous thrombosis. We performed an additional study on the hemostatic capacity of the patient as she was relatively young and had no risk factors for thrombosis. She was negative for lupus anticoagulant, and had normal homocysteine (9.1 nmol/ml) level, normal protein S antigen level of 112% (reference value 60%-150%), normal protein S activity of 79% (reference value 67%-164%), and normal protein C antigen level of 90% (reference value 70%-150 %), while she had a low protein C activity of 50% (reference value 64%-146%). Based on laboratory data, she was diagnosed with protein C deficiency type Ⅱ. She received a protein C gene test after providing consent (approved by the Ethics Review Committee of this hospital, approval number R20-027, approval date June 24, 2020), because PC activity may decrease for various reasons, such as infections and liver diseases. As a result of the direct sequence method, a heterozygous mutation in exon 7c. 577-579delAAG, p.Lys193del on protein C was found, which was considered to be cerebral vein sinus thrombosis due to hereditary protein C deficiency (Figure 2).

No genetic testing was performed on her family. Anticoagulant therapy with heparin was started on the third day after admission, and oral administration of dabigatran was started on the 17th day after admission. On the 22nd day, she was discharged. As a result of treatment, she had no thromboembolic events or other complications within six months.

## Discussion

Here, we reported a rare case of cerebral venous thrombosis caused by hereditary protein C deficiency that was treated with dabigatran in the chronic phase. In our case, the patient presented with involuntary movements and seizures in her right upper and lower limbs. Magnetic resonance venography confirmed cerebral venous thrombosis. In addition, a protein C activity test and genetic analysis revealed the complication of hereditary protein C deficiency. Dabigatran was administrated in the chronic phase and prevented complications and relapse of thromboembolism. Cerebral venous thrombosis is an uncommon and serious disease that presents with various neurological symptoms. Many cases of cerebral venous thrombosis have been linked to inherited and acquired thrombophilia, pregnancy, infection, and malignancies. Among them, the screening for thrombophilia is especially needed, because patients with thrombophilia need to extend the treatment period of anticoagulants [1]. In addition, patients with protein C deficiency have another problem with warfarin-induced skin necrosis [8]. Protein C has a short half-life among hemostatic-related proteins of vitamin K-dependent proteins [9]. Thus, warfarin induces a transient hypercoagulable state, causing vascular occlusion and tissue necrosis. In addition, genetic testing may be important for accurate diagnosis and prevention of unnecessary complications of anticoagulant treatment, because protein C deficiency type II occurs secondary to infections and cirrhosis [9,10]. In our case, the hemostatic capacity screening of patients with cerebral venous thrombosis made a diagnosis of protein C deficiency type II, and the patients had the c.577-579delAAG, p.Lys193del (heterozygotes) mutation in the protein C gene exon7. Genetic mutations have been identified in thrombosis in the deep veins of the leg cases [11] but have not been reported in cerebral venous thrombosis cases. The treatment of cerebral venous thrombosis in the chronic phase involves anticoagulant therapy to prevent recurrence of cerebral venous thrombosis and development of extracerebral venous thrombosis, such as deep venous thrombosis [1]. Warfarin is the most commonly used anticoagulant drug but carries certain risks, including a high rate of thromboembolic and bleeding complications, a requirement for routine monitoring, a requirement for careful dosing, and specific complications in patients with protein C deficiency, such as warfarin-induced skin necrosis [8,12]. In addition, a case of recurrent cerebral venous thrombosis with hereditary protein C deficiency in patients who received warfarin was reported [13]. A randomized control trial revealed that direct oral anticoagulants, such as dabigatran, appear to have similar effectiveness and safety compared to warfarin for preventing recurrent cerebral venous thrombosis [14]. Dabigatran may solve many of the problems associated with warfarin, including protein C deficiency-specific complications [15]. In our case, the patient received dabigatran as anticoagulant therapy in the chronic phase and developed no thromboembolic events or other complications.

## Conclusions

We presented a case of cerebral venous thrombosis caused by hereditary protein C deficiency who was administered dabigatran in the chronic phase. Our case indicated that dabigatran might be one of the alternative choices for patients complicated with cerebral venous thrombosis and protein C deficiency.
